# Structure-based identification and characterisation of structurally novel human P2X7 receptor antagonists

**DOI:** 10.1016/j.bcp.2016.07.020

**Published:** 2016-09-15

**Authors:** Emily A. Caseley, Stephen P. Muench, Colin W. Fishwick, Lin-Hua Jiang

**Affiliations:** a School of Biomedical Sciences, Faculty of Biological Sciences, University of Leeds, UK; b School of Chemistry, University of Leeds, UK

**Keywords:** zfP2X4R, zebrafish P2X4 receptor, P2X7R, P2X7 receptor, hP2X7R, human P2X7R, rP2X7R, rat P2X7R, BzATP, 2,3-O-(4-benzoylbenzoyl)-ATP, PI, propidium iodide, BBG, brilliant blue G, TNP-ATP, trinitrophenyl-ATP, HEK293 cells, human embryonic kidney 293 cells, hP2X7R antagonists, Virtual screening, Ca^2+^ imaging, Patch-clamp recording, YO-PRO-1 dye uptake

## Abstract

The P2X7 receptor (P2X7R) plays an important role in diverse conditions associated with tissue damage and inflammation, meaning that the human P2X7R (hP2X7R) is an attractive therapeutic target. The crystal structures of the zebrafish P2X4R in the closed and ATP-bound open states provide an unprecedented opportunity for structure-guided identification of new ligands. The present study performed virtual screening of ∼100,000 structurally diverse compounds against the ATP-binding pocket in the hP2X7R. This identified three compounds (C23, C40 and C60) out of 73 top-ranked compounds by testing against hP2X7R-mediated Ca^2+^ responses. These compounds were further characterised using Ca^2+^ imaging, patch-clamp current recording, YO-PRO-1 uptake and propidium iodide cell death assays. All three compounds inhibited BzATP-induced Ca^2+^ responses concentration-dependently with IC_50_s of 5.1 ± 0.3 μM, 4.8 ± 0.8 μM and 3.2 ± 0.2 μM, respectively. C23 and C40 inhibited BzATP-induced currents in a reversible and concentration-dependent manner, with IC_50_s of 0.35 ± 0.3 μM and 1.2 ± 0.1 μM, respectively, but surprisingly C60 did not affect BzATP-induced currents up to 100 μM. They suppressed BzATP-induced YO-PRO-1 uptake with IC_50_s of 1.8 ± 0.9 μM, 1.0 ± 0.1 μM and 0.8 ± 0.2 μM, respectively. Furthermore, these three compounds strongly protected against ATP-induced cell death. Among them, C40 and C60 exhibited strong specificity towards the hP2X7R over the hP2X4R and rP2X3R. In conclusion, our study reports the identification of three novel hP2X7R antagonists with micromolar potency for the first time using a structure-based approach, including the first P2X7R antagonist with preferential inhibition of large pore formation.

## Graphical abstract

**Figure:**
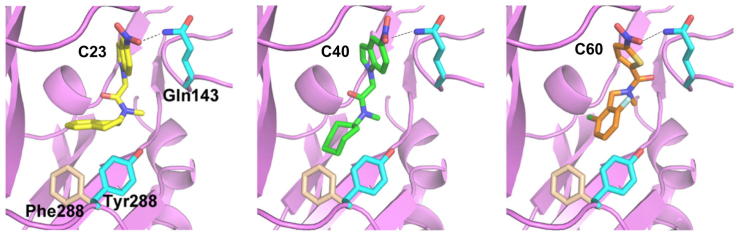

## 1 Introduction

P2X receptors are a family of trimeric protein complexes that function as ATP-gated calcium-permeable, nonselective cationic channels [32]. The P2X7 receptor (P2X7R), the last identified member of the P2X receptor family, acts as an ion channel during brief stimulation. However, prolonged receptor activation can induce the formation of large pores which allow the passage of molecules of up to 900 Da in size which ultimately results in cell death. Because of this, the P2X7R is known as a cytolytic receptor [42]. The P2X7R is found in almost all tissues of the body, being expressed in particular in cells of haematopoietic origin [22]. It plays an important role in a diversity of conditions associated with tissue damage or inflammation including chronic pain, rheumatoid arthritis and age-related macular degeneration, making the P2X7R an attractive therapeutic target [33], [38], [1]. Huge drug discovery efforts over the past few years have led to the discovery of numerous P2X7R antagonists [17], [41], [27]. Whilst clinical trials of the first two P2X7R antagonists against rheumatoid arthritis showed disappointing outcomes [26], [39], a more recent clinical trial shows promise in the therapeutic use of a P2X7R antagonist (AZD9056) in the treatment of moderate-to-severe Crohn’s disease [10].

In order to exploit the full potential of P2X7R antagonists as therapeutics, further efforts are inevitably required to develop more cost-effective and efficient drug discovery approaches. These will help to expand the structural diversity of P2X7R antagonists as well as to develop tools which will help us better understand the role of the P2X7R in disease mechanisms, particularly its large pore formation [37], [43], [14]. None of the known antagonists can definitively discriminate between the ion channel and large pore-forming functionalities of the P2X7R. Furthermore, all the currently available P2X7R antagonists have been discovered by high-throughput screening which involves the random testing of large compound libraries against the receptor; a process that is often time-consuming, costly and inefficient. A relatively recent breakthrough in the study of P2X receptors is the determination of the atomic structures of the zebrafish P2X4R (zfP2X4R) in both the apo, closed, and ATP-bound, open, states [18]. Homology models of the P2X7R have been useful in providing structural insights into the effects of disease-associated mutations on P2X7R function [7], [35], [2], [24] and the striking species difference of P2X7R antagonists [6]. The key residues coordinating ATP binding identified in the ATP-bound zfP2X4R structure are almost completely conserved through the mammalian P2X receptors [4] but not all the residues participating in the formation of the ATP-binding pocket are identical [24]. The ATP binding pocket in itself is unconventional, not consisting of a well-known ATP binding motif such as the Walker motif. As such, this region is a favourable target for therapeutic compounds due to its reduced likelihood of interactions with other vital ATP-binding proteins. Such structural features offer a feasible opportunity permitting the structure-based identification of small molecules which bind to this ATP-binding pocket with receptor-subtype specificity. In the present study, we used a sphere 10 Å in diameter encompassing the ATP-binding pocket in the hP2X7R homology model in the virtual screening of a structurally diverse compound database. In combination with functional assays, we have identified three structurally novel hP2X7R antagonists, including the first hP2X7R antagonist which displays selective inhibition of the large pore formation without having an effect on the ion channel function.

## 2 Materials and methods

### 2.1 Chemicals

General chemicals used in the study, ATP, 2,3-O-(4-benzoylbenzoyl)-ATP (BzATP), trinitrophenyl-ATP (TNP-ATP), brilliant blue G (BBG), YO-PRO-1 iodide, propidium iodide (PI) and G418 were purchased from Sigma (Dorset, UK). AZ11645373 and 5-BDBD were from Tocris Bioscience (Bristol, UK). All 73 compounds tested in the study (C1-C73) were sourced from Enamine (Ukraine) with a purity of ⩾93% determined by high performance liquid chromatography analysis.

### 2.2 Homology modelling

Structural models of the human and rat P2X7R were produced based on the atomic structures of the zfP2X4R in the apo, closed state and ATP-bound open state (Protein Data Bank code 4DW0 and 4DW1, respectively) using Modeller version 9.12 [11], as described in our previous studies [21], [6]. In brief, one hundred versions of the P2X7R were generated for each model and the five with the lowest energy were analysed using MolProbity [8]. Those with the greatest percentage of residues in allowed regions of the Ramachandran plot were selected for use in further investigations. The non-conserved loop region between the β2 and β3 strands was modelled de novo using the ModLoop server [13].

### 2.3 Virtual screening

Virtual screening was carried out using eHiTS version 12 [45]. The ATP-binding pocket file used in the screening was produced in SPROUT [16] and consisted of a sphere 10 Å in diameter centred on the bound ATP molecule. The ZINC12 database, containing approximately 100,000 structurally diverse compounds [20], were docked to this ATP-binding pocket. 500 of the best eHiTS scoring compounds were further scored using SPROUT. 42 of the 50 compounds (C1–C42) ranked with the highest predicted energy binding scores were commercially available and were tested in the initial functional assay. Further screening of the ZINC12 database was performed based on structural similarity using the ZINC12 website search function in order to carry out ‘structure-activity relationships by catalogue’ against the common shapes and functional groups of the two hits (C23 and C40). The top 31 compounds (C43–C73) with >80% structural similarity were tested in functional assays. PyMOL [9] was used for the visual inspection of ligand-receptor interactions and later graphical representations.

### 2.4 Cell culture

Human embryonic kidney (HEK) 293 cells stably expressing C-terminally EE-tagged human or rat P2X7R [42], [34], human P2X4R [15] and rat P2X3R [28] were generated in previous studies. A stable HEK293 cell line expressing C-terminally His-tagged human P2X7R was generated in this study and there was no difference in the experimental results using EE-tagged and His-tagged human P2X7R. These cells were maintained in Dulbecco’s Modified Eagle Medium (Life Technologies) supplemented with 10% foetal bovine serum and 200 ng/ml G418 in a tissue culture incubator at 37°C and 5% CO_2_ under humidified conditions. Cells were cultured in T25 flasks and split (every 3–4 days) once confluent.

### 2.5 Calcium measurements

FlexStation II and III (Molecular Devices) were used to measure intracellular Ca^2+^ concentrations ([Ca^2+^]_i_). Human or rat P2X7R-expressing HEK293 cells were plated in poly-d-lysine-coated 96-well plates (Molecular Devices) with 50,000 cells per well 24 h prior to use. Cells were rinsed with standard buffer solution (SBS) containing: 134 mM NaCl, 5 mM KCl, 1.2 mM MgCl_2_ and 1.5 mM CaCl_2_, 8 mM glucose, 2.4 mM HEPES, pH 7.4. After 100 μl of loading buffer consisting of SBS containing 1 μM Fura-2/AM (Life Technologies) and 0.01% (v:v) pluronic acid (ThermoFisher) was added to each well, the plate was incubated at 37 °C for 45 min. Cells were rinsed once with SBS, after which 160 μl of SBS containing either 0.01–0.02% dimethyl sulphoxide (DMSO) used to prepare compound stock solutions or test compound were added to each well. The plate was incubated at 37 °C for 30 min. Cells were excited at 340 nm and 380 nm alternatively and the emission at 510 nm recorded using a FlexStation. The ratio of fluorescence intensity (F340/F380) was used to indicate the [Ca^2+^]_i_. The basal [Ca^2+^]_i_ was recorded for 60 s prior to the exposure to agonist and recording was continued for a further 120 s. BzATP at 300 μM was used to evoke maximal activation of human and rat P2X7R (e.g. [3]), and ATP at 100 μM at the hP2X4R and rP2X3R [28], [15]. The maximal changes in F340/F380 (ΔF340/380) were used for quantitative analysis.

### 2.6 Patch-clamp recording

Membrane ionic currents were measured using patch-clamp recording in the whole-cell configuration at room temperature using an Axopatch 200B amplifier and analysed with pClamp 10.3 software (Axon Instruments) as described in our previous study [2]. In brief, HEK293 cells expressing the hP2X7R were seeded onto 10-mm glass cover slips prior to use. The cover slip was placed in a recording chamber connected to a solution exchange system driven by a gravity feed at a rate of 5 ml min^−1^. Patch microelectrodes with a resistance of approximately 1–5 MΩ were produced using borosilicate glass capillaries (World Precision Instruments). Cells were kept at a holding potential of −80 mV. Agonists and antagonists were applied using a rapid solution changer (RSC-160, Biologic Science Instruments). The standard extracellular solution contained: 147 mM NaCl, 2 mM KCl, 1 mM MgCl_2_, 2 mM CaCl_2_, 10 mM HEPES, and 13 mM glucose. The intracellular solution contained: 145 mM NaCl, 10 mM EDTA and 10 mM HEPES. Divalent cations are known to strongly inhibit the P2X7R [42] and thus in a majority of experiments agonist-induced currents were recorded in low divalent extracellular solution containing: 147 mM NaCl, 2 mM KCl, 0.3 mM CaCl_2_, 10 mM HEPES and 22 mM glucose. All solutions were adjusted to pH 7.3 with 5 M NaOH. The currents were evoked by 4 s application of 300 μM BzATP with an interval of 2–4 min and the peak currents were used for quantitative analysis as described in our previous studies [3].

### 2.7 YO-PRO-1 uptake assay

YO-PRO-1 uptake was recorded mainly using FlexStation as described in a previous study [5]. HEK293 cells expressing the hP2X7R were seeded in poly-d-lysine coated 96-well plates as described above for Ca^2+^ measurements. The assay buffer comprised: 147 mM NaCl, 2 mM KCl, 0.3 mM CaCl_2_, 10 mM HEPES, and 22 mM glucose, pH 7.3. Cells were washed with assay buffer and incubated at 37 °C for 20 min with 80 μl of assay buffer containing 1 μM YO-PRO-1 with either DMSO or the test compound. Cells were excited at 485 nm and the emission recorded at 530 nm. Recordings lasted 120 s and BzATP was added at 20 s with a final concentration of 300 μM which is known to induce maximal P2X7 receptor-dependent dye uptake [25], [3]. The maximal changes in F530 were used for quantitative analysis. Single cell imaging was also used to examine the effect of C60 on ATP-induced hP2X7R-dependent dye uptake. Cells were plated in 24-well plates with 20,000 cells per well 24 h prior to use. 1 mM ATP alone or with 5 μM C60 and 10 μM C10 as well as YO-PRO-1 at a final concentration of 1 μM were added into the cell culture media and incubated at 37 °C for 30 min. Cells were imaged using an EVOS FL cell imaging system (Life Technologies). For each well, YO-PRO-1 stained cells were counted in one randomly selected field and presented as the percentage of the total number of cells identified by DAPI staining in the same field. Cell counting was carried out using ImageJ.

### 2.8 PI staining cell death assay

PI staining was used to assess cell death as described in our previous study [29] in cells treated with ATP, a condition that is similar to P2X7 receptor-mediated cell death under pathological conditions such as tissue damage and inflammation. Cells were plated in six well-plates with 40,000 cells per well 24 h prior to use. ATP at 3 mM alone or together with the test compounds at the indicated concentrations were added to the culture media and cells were incubated at 37 °C and 5% CO_2_ for 6 h. PI was added with a final concentration of 5 μg/ml and cells were incubated for a further 30 min. Cells were counterstained with DAPI. Images were captured using an EVOS FL cell imaging system (Life Technologies). For each well, PI-stained cells were counted in one randomly selected field and presented as the percentage of the total number of cells identified by DAPI staining in the same field. Cell counting was carried out using ImageJ.

### 2.9 Data presentation and analysis

All results, where appropriate, are presented as mean ± standard error of the mean (SEM). The antagonist concentrations evoking half inhibition (IC_50_) were derived using Origin to fit the data from each independent experiment (Ca^2+^ response and YO-PRO-1 uptake) or cells (current) to the Hill equation: *I* = 100/(1 + ([B]/IC_50_)^n^), where *I* is the BzATP-induced responses (Ca^2+^ response, current or YO-PRO-1 uptake) following exposure to identified concentrations of antagonist ([B]) and expressed as the percentage of the control responses and *n* is Hill coefficient. The solid line shown in the figures represent the fitting of mean data. Statistical analysis was carried out using Origin. Student’s *t*-test was used for two groups and one-way analysis of variance test and Tukey’s *post hoc* test for more than two groups, and the difference was considered to be significant at *p* < 0.05.

## 3 Results

### 3.1 Identification of novel hP2X7R antagonists by structure-based virtual screening

**Fig. 1:**
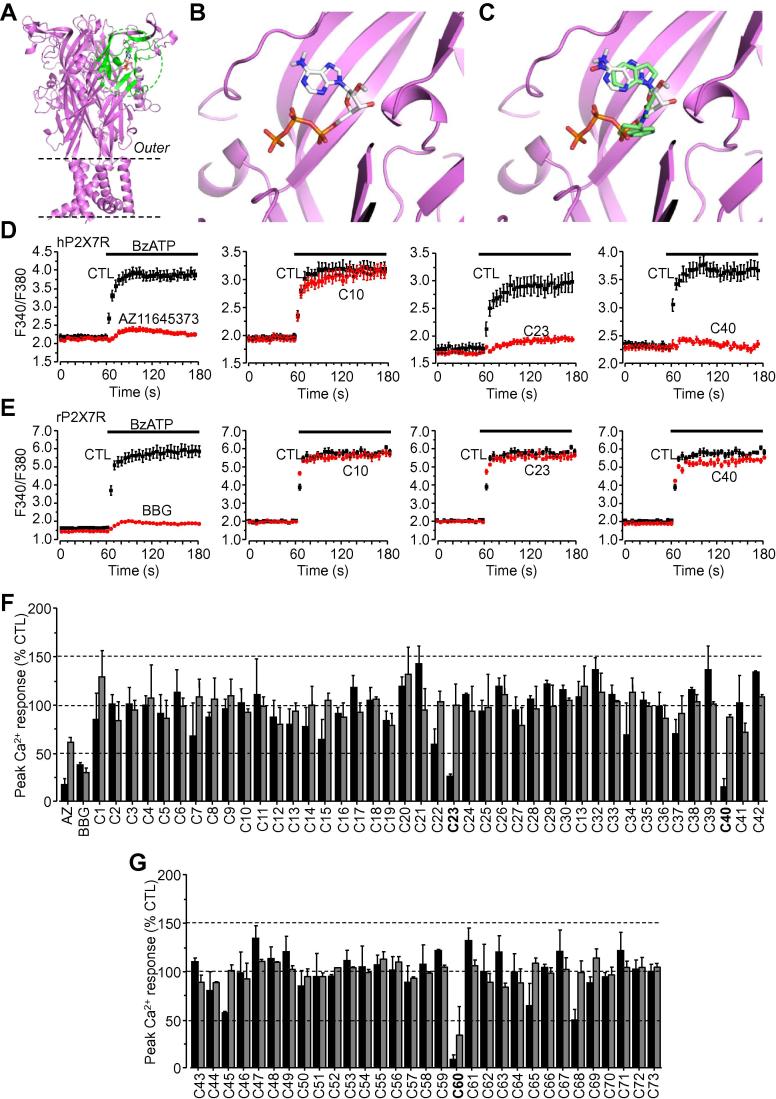
Three compounds identified from virtual screening of the ZINC12 database that inhibit the hP2X7R. **A.** The trimeric hP2X7R homology model in the closed state, with an ATP molecule docked to one of the three inter-subunit ATP-binding pockets. The 10 Å sphere ATP-binding pocket centred on the bound ATP molecule is highlighted in green. **B.** The predicted ATP binding conformation within the ATP-binding pocket in the hP2X7R. **C.** Docking of compound C23 (green) from the ZINC12 compound library in the ATP-binding pocket in the hP2X7R and ATP (silver) shown for comparison. **D.** The predicted binding BzATP conformation within the ATP-binding pocket in the hP2X7R. **E**–**F**. Representative Flex-Station recordings of Ca^2+^ responses induced by 300 μM BzATP in HEK293 cells expressing the hP2X7R (E) and rP2X7R (F), with the control responses shown in black and the responses in cells treated with compound C10, C23 or C40 at 10 μM in red. 200 nM AZ11645373 and 10 μM BBG were used as the positive control inhibiting the human and rat P2X7R respectively. **G.** Summary of the effects of 42 compounds on BzATP-induced Ca^2+^ responses mediated by the hP2X7R shown in black and the rP2X7R in grey. Results were from 8 to 12 wells of cells from 3 independent experiments. **H**. Summary of the effects of 31 compounds with structural similarity to C23 and C40 on BzATP-induced Ca^2+^ responses mediated by the hP2X7R shown in black and the rP2X7R in grey. Results were from 8 to 12 wells of cells from 3 to 5 independent experiments. (For interpretation of the references to colour in this figure legend, the reader is referred to the web version of this article.)

Comparison of the ATP-bound zfP2X4R structure and the hP2X7R homology model reveals 62% amino acid sequence identity between these receptors within the ATP-binding pocket in a 10 Å sphere centred on the bound ATP molecule (Fig. 1A–B). The essential nature of the ATP binding pocket and its non-traditional fold in the hP2X7R made it a suitable target to perform virtual screening of the ZINC12 database, which contains approximately 100,000 structurally diverse compounds [20]. Each compound was docked individually into the ATP-binding pocket using eHiTS (e.g., Fig. 1C) with a corresponding predicted energy binding score being calculated. For the 50 compounds with the highest scores, 42 were commercially available and had predicted energy binding scores ranging from −4 to 10.5 kcal/mol. These values are comparable to those predicted for known hP2X7R antagonists including AZ11645373 (−10.8 kcal/mol), SB203580 (−8.75 kcal/mol) and KN-62 (−5.2 kcal/mol) as reported in our recent study [6]. To initially test the top 42 compounds we applied the compounds at 10 μM to determine their effects on Ca^2+^ responses in HEK293 cells expressing hP2X7R induced by 300 μM BzATP, a structural analogue of ATP which is more potent than ATP at the P2X7R and is predicted to bind to the ATP-binding site (data not shown). None of the compounds showed detectable agonist activity. Two compounds, ZINC67825876 (C23 from here onward) and ZINC58368839 (C40), inhibited BzATP-induced Ca^2+^ responses by 73.2 ± 2% and 84.3 ± 7% respectively, whilst all other compounds had no or modest effect, as illustrated by ZINC19868610 (C10) (Fig. 1D and F). The inhibition by C23 and C40 was similar to that by BBG (71.5 ± 5%) and AZ11645373 (81.9 ± 5%) (Fig. 1D and F). These 42 compounds were also tested against BzATP-induced Ca^2+^ responses in HEK293 cells expressing the rP2X7R (Fig. 1E and G). BBG was used as a positive control and strongly inhibited BzATP-induced Ca^2+^ responses, whereas AZ11645373 was far less effective (Fig. 1F). None of the compounds caused significant inhibition of the rP2X7R, including C23 and C40 (Fig. 1E and F). Examination of C23 and C40 reveals noticeable similarities as well as substantial differences in their structures (Table 1). A number of additional compounds with a high level of structural similarity (⩾80%) were identified from the ZINC12 database using the ZINC12 website search function. The top 31 compounds from this new search were tested at 10 μM against the human and rat P2X7R using FlexStation measurements of BzATP-induced Ca^2+^ responses. ZINC09315614 (C60) almost completely ablated BzATP-induced Ca^2+^ responses in hP2X7R-expressing cells (91.2 ± 4%), and also significantly but less effectively attenuated BzATP-induced Ca^2+^ responses in rP2X7R-expressing cells (66.2 ± 22%) (Fig. 1G). These results show that the application of a structure-based approach by combining structural homology modelling, virtual screening and functional assays enabled the identification of C23, C40 and C60, which represent 3 out of a total of 73 compounds tested, and cause strong inhibition of the hP2X7R.

### 3.2 Further pharmacological characterisation of C23, C40 and C60

**Fig. 2:**
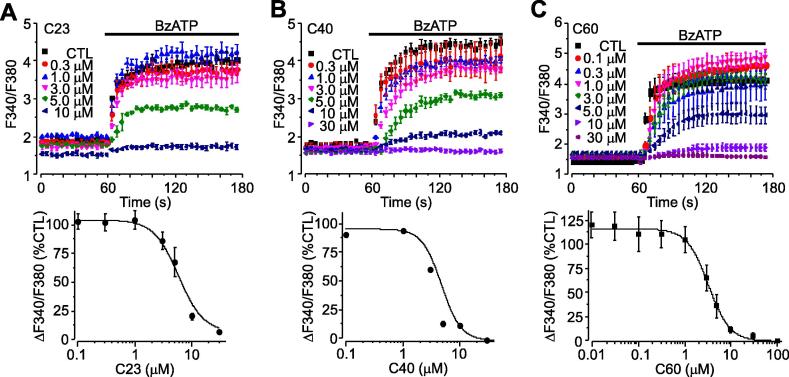
Concentration-dependent effects of C23, C40 and C60 on BzATP-induced Ca^2+^ responses in hP2X7R-expressing HEK293 cells. *Top*, representative Ca^2+^ responses induced by 300 μM BzATP in control cells or cells treated with C23 (A), C40 (B) and C60 (C) at the indicated concentrations. *Bottom*, concentration-Ca^2+^ response relationship curves with the solid lines representing the fit of the mean data to Hill equation. Each data point represents the mean from 8 to 12 wells of cells from 3 to 5 independent experiments.

**Fig. 3:**
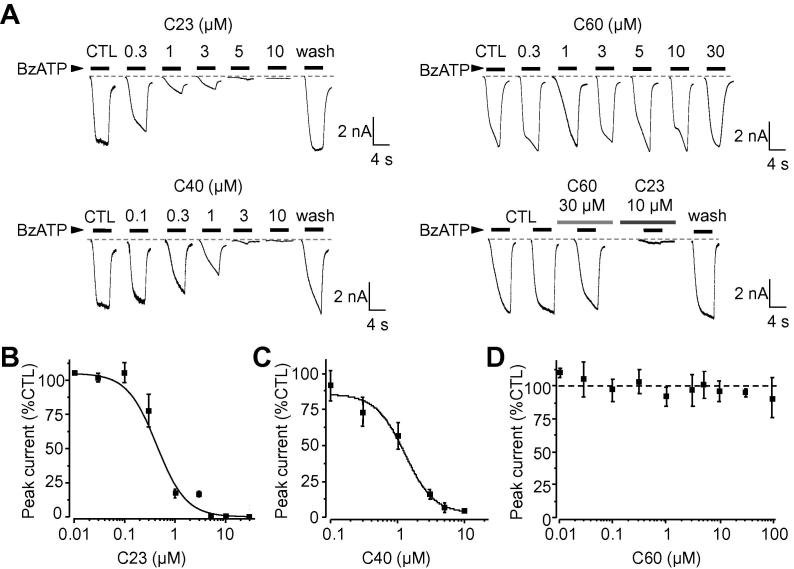
Concentration-dependent effects of C23, C40 and C60 on BzATP-induced currents in hP2X7R-expressing HEK293 cells. **A.** Representative recordings of currents induced by 4 s application of 300 μM BzATP in cells before (CTL) and after exposure to C23, C40 and C60 at the indicated concentrations. Note that the current inhibition by C23 and C40 was reversed upon washing, and the current was not inhibited by C60 but abolished by subsequent application of C23. **B**–**D.** Concentration-current response relationship curves for C23 (B), C40 (C) and C60 (D). The solid lines in B and C represent the fit of the mean data to the Hill equation. Each data point represents mean from 4 cells from 3 to 4 independent experiments.

The potency of C23, C40 and C60 when inhibiting the hP2X7R was further characterised by recording BzATP-induced Ca^2+^ responses and currents in HEK293 cells expressing the hP2X7R. The FlexStation and patch-clamp recording were used respectively, as these methods represent the most commonly used techniques in the study of ion channel pharmacology. As illustrated in Fig. 2, these three compounds inhibited Ca^2+^ responses induced by 300 μM BzATP in a concentration-dependent manner, with complete inhibition at the highest concentrations applied (10–30 μM). Fitting the concentration-response relationship curves to the Hill equation yielded IC_50_ values of 5.1 μM ± 0.3 μM (n = 6), 4.8 ± 0.8 μM (n = 6) and 3.2 ± 0.2 μM (n = 6) for C23, C40 and C60, respectively (Fig. 2A–C). Similarly, C23 and C40 concentration-dependently inhibited BzATP-induced currents, with IC_50_ values of 0.35 ± 0.3 μM (n = 6) and 1.2 ± 0.1 μM (n = 6) respectively (Fig. 3A–C). Current inhibition was complete and readily reversed upon washing (Fig. 3A). Surprisingly, despite exhibiting similar potency as C23 and C40 in inhibiting BzATP-induced Ca^2+^ responses, C60 caused no inhibition of BzATP-induced currents even when applied at 30–100 μM (Fig. 3A and D). BzATP-induced currents in the same current recordings were abolished by the subsequent application of C23 (Fig. 3A).

**Fig. 4:**
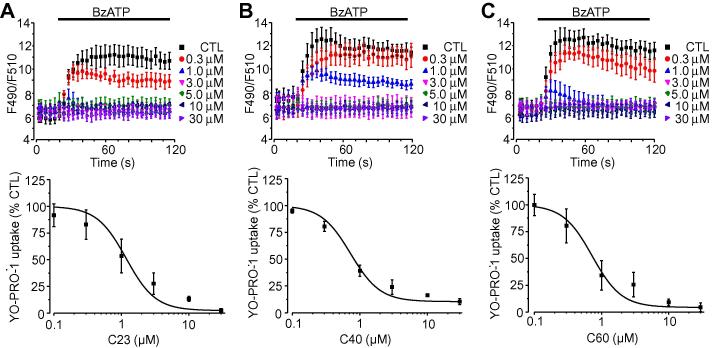
Concentration-dependent effects of C23, C40 and C60 on BzATP-induced large pore formation in hP2X7R-expressing HEK293 cells. *Top*, representative recording of 300 μM BzATP induced YO-PRO-1 uptake in control cells (CTL) or cells with treated with C23 (A), C40 (B) and C60 (C) at the indicated concentrations. *Bottom*, concentration-dye uptake relationship curves with the solid lines representing the fit of the mean data to Hill equation. Each data point represents the mean from 4 to 6 wells of cells from 3 to 4 independent experiments.

As introduced above, prolonged P2X7R activation can induce large pore formation. This can be visualised under physiological conditions by monitoring the uptake of fluorescent dyes with large molecular weights such as YO-PRO-1 (375 Da) [44]. C23, C40 and C60 were further investigated to see if they inhibited P2X7R-dependent large pore formation using FlexStation to measure YO-PRO-1 uptake in hP2X7R-expressing HEK293 cells. C23, C40 and C60 concentration-dependently inhibited YO-PRO-1 uptake induced by 300 μM BzATP, with IC_50_ values of 1.8 ± 0.9 μM (n = 12), 1.0 ± 0.1 μM (n = 12) and 0.8 ± 0.2 μM (n = 16), respectively (Fig. 4A–C). We also used single cell imaging to examine the effect of C60 on ATP-induced hP2X7R-dependent dye uptake. The percentage of YO-PRO-1 positive cells was increased by >4 times upon treatment with 1 mM ATP for 30 min (14.7 ± 4.0% and 63.0 ± 11.7% in control cells and ATP-treated cells, n = 3, p < 0.01). The percentage of YO-PRO-1 positive cells in ATP-treated cells was significantly reduced by treatment with 5 μM C60 (33.8 ± 5.6%, n = 3, p < 0.001) but not affected by treatment with 10 μM C10 (69.2 ± 3.2%, n = 3; p > 0.05). These results provide further evidence to support selective inhibition by C60 of hP2X7R-dependent large pore formation.

Taken together, these results provide clear evidence to show that C23 and C40 inhibit both hP2X7R ion channel activation and large pore formation but C60 preferentially targets large pore formation without effect on the ion channel function.

### 3.3 Effects of C23, C40 and C60 on other P2XRs

**Fig. 5:**
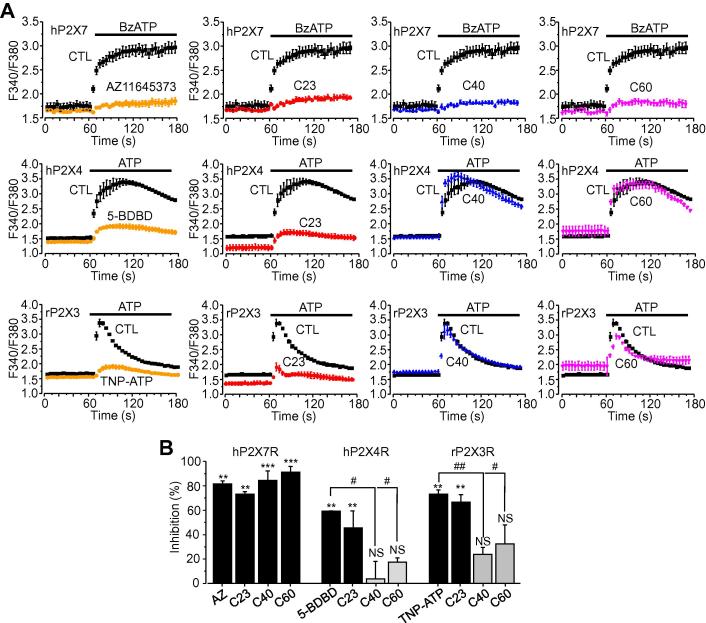
Effects of C23, C40 and C60 on agonist-induced Ca^2+^ responses in HEK293 cells expressing different P2X receptors. **A.** Representative recording of Ca^2+^ responses in HEK293 cells expressing hP2X7R, hP2X4R and rP2X3R evoked by 100 μM BzATP and 100 μM ATP respectively in the absence and presence of 10 μM C23, C40 C60, AZ116435373, 5-BDBD and TNP-ATP. Note the distinct kinetics of the Ca^2+^ responses in cells expressing different receptors. **B.** Summary of percentage inhibition of the compounds as indicated. Results are presented as the mean from 8 to 12 wells of cells from 3 to 5 independent experiments. **, p < 0.01; ***, p < 0.005; NS, no significant difference compared to control. #, p < 0.05; ##, p < 0.01 compared to the inhibition by positive control antagonists AZ116435373 at the hP2X7R, 5-BDBD at the hP2X4R or TNP-ATP at the rP2X3R.

C23, C40 and C60 were examined next for their specificity by determining their effects on the other members of the P2X receptor family, particularly the hP2X4R. The P2X4R has the highest level of sequence identity with the P2X7R over the whole receptor and also at the ATP-binding pocket used in virtual screening and, furthermore, the two receptors co-exist in many cell types. C23 applied at 10 μM strongly reduced 100 μM ATP-induced Ca^2+^ responses in hP2X4R-expressing HEK293 cells in a manner comparable to the P2X4R antagonist 5-BDBD (Fig. 5A and B). However, both C40 and C60 also applied at 10 μM resulted in no significant effect (Fig. 5A and B). The three compounds were further tested in rP2X3R-expressing HEK293 cells, in which 100 μM ATP induced Ca^2+^ responses with transient kinetics (Fig. 5A) due to the strong desensitisation characteristic of this receptor [32]. C23 was also effective at 10 μM in suppressing rP2X3R-mediated ATP-induced Ca^2+^ responses in a manner similar to the P2X3R antagonist TNP-ATP (Fig. 3A and B). Both C40 and C60 caused smaller but statistically insignificant inhibition (Fig. 5A and B). Therefore, whilst C23 is largely indiscriminate, both C40 and C60 exhibit much greater specificity towards the hP2X7R.

### 3.4 Inhibition of P2X7R-mediated cell death

**Fig. 6:**
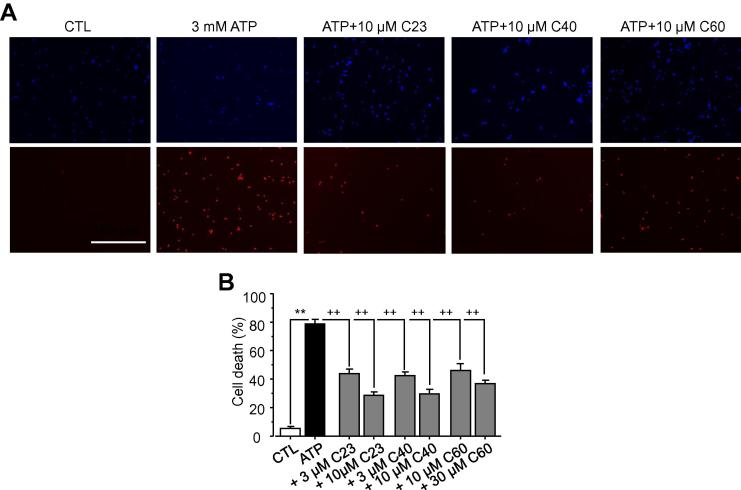
Effects of C23, C40 and C60 on ATP-induced cell death in hP2X7-expressing HEK293 cells. **A.** Representative fluorescent images showing all cells identified by DAPI staining and PI-stained dead cells under the conditions indicated. Cells were treated with 3 mM ATP alone or together with compounds at the indicated concentrations for 6 h, and untreated cells were used as a control (CTL). Scale bar is 100 μm. **B.** Summary of percentage of cell death under the indicated conditions. Results are presented as the mean from 12 independent experiments. **, p < 0.01 compared to control. ++, p < 0.05 compared to cells treated with alone.

The P2X7R is a well-known cytolytic receptor which plays a critical role in mediating ATP-induced cell death in vivo under pathological conditions as well as under in vitro experimental conditions, a unique functional role which distinguishes the P2X7R from all the other P2X receptors [22]. As such, inhibition of the P2X7R offers protection against ATP-mediated cell death in a number of cells expressing the P2X7R [12], [30], [36]. Therefore, C23, C40 and C60 compounds were finally tested for their effectiveness in preventing ATP-induced cell death in hP2X7R-expressing HEK293 cells to provide further supporting evidence for their action on the hP2X7R. Exposure of cells to 3 mM ATP for 6 h caused cell death by 74 ± 7% (n = 12) as determined by PI staining assays (Fig. 6A and B). Pre-treatment with C23 at 3 and 10 μM, C40 at 3 and 10 μM or C60 at 10 and 30 μM strongly reduced ATP-induced cell death by approximately or >50% (Fig. 6A and B). These results provide further evidence to support the notion that compounds identified in this study inhibit the hP2X7R.

## 4 Discussion

Studies over the past decade have revealed a crucial role for the P2X7R in a range of debilitating disease conditions [23]. Emerging evidence supports the hP2X7R as a druggable target [10]. In order to exploit the full potential of hP2X7R antagonists as therapeutics, more efforts are required to increase the structural diversity of hP2X7R antagonists as well as to better understand the underlying disease mechanisms. All currently known P2X7R antagonists have been identified through the random functional screening of large compound libraries, which can be time-consuming, costly and inefficient. Using virtual screening of a large set of compounds against the hP2X7R ATP-binding pocket followed by functional assays, the present study identified two hits (C23 and C40) (Fig. 1D and F). A further 31 compounds were generated based on structural similarities to C23 and C40 which identified a third compound, C60 (Fig. 1G). Detailed functional characterisations, by determining their effects on hP2X7R-mediated Ca^2+^ responses, currents and YO-PRO-1 uptake, consistently indicate micromolar potency at the hP2X7R, albeit with some variations that are likely related to the functional assays used (Fig. 2, Fig. 3, Fig. 4). Moreover, all three compounds effectively protected against ATP-induced cell death (Fig. 6), providing further evidence to support that they antagonise the hP2X7R. Subtype specificity is of great importance when using antagonists in the study of the role of the hP2X7R in ATP-induced physiological functions and diseases as well as for the development of therapeutic compounds. Of these three compounds, C40 and C60 exhibited significant specificity towards the hP2X7R over the other P2X receptors examined (Fig. 5), including the hP2X4R which shares the highest level of sequence identity with the hP2X7R. Thus, it is unlikely but remains to be confirmed that C40 and C60 cause potent inhibition at the other P2X receptors with less structural similarity to the hP2X7R. Evidently, structure-activity relationship studies are required for structural optimisation of these compounds in order to further improve their potency and specificity. Nonetheless, the present study is the first to identify structurally novel P2X7R antagonists using a structure-based approach, which can be more time-saving and cost-effective than the conventional screening approach.

It has been well-documented that prolonged or repeated activation of the P2X7R induces large pore formation, but it still remains a subject of debate if this is through P2X7R itself or via the activation of a distinctive protein which forms the large pore [32], [44], [21]. Regardless of the underlying mechanisms, recent studies show that P2X7R-dependent large pore functionality is of high relevance to the pathogenesis of diseases such as chronic pain [37], osteoporosis [43], and aged-related macular degeneration responsible for the loss of vision in elderly people [14]. Emerging evidence supports that blocking this pore is a valuable therapeutic intervention [14]. None of the currently available P2X7R antagonists effectively discriminate between the ion channel and large pore functions. In this regard, C60 is of interest as it is the first reported P2X7R antagonist that selectively prevents large pore formation (Fig. 4) without affecting the ion channel function (Fig. 3). C60 will be useful as a pharmacological tool to better understand large pore formation and its role in P2X7R-mediated physiological functions and diseases. It may also act as a potential lead compound for development of therapeutics targeting this pivotal pore-forming function.

**Fig. 7:**
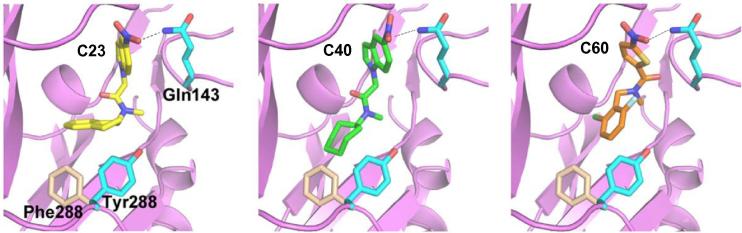
Proposed structural basis for ligand-receptor interactions and species difference for C23, C40 and C60. The three hit compounds (from left to right: C23, C40 and C60) are docked to the ATP-binding pocket in the structure homology models of hP2X7R and rP2X7R. Assessments of the ligand-receptor interactions predict that Tyr288 and Gln143 in the hP2X7R participate in interactions with the bulky ring structure and hydrogen bonding with the NO_2_ group in the compounds, respectively. Whilst Tyr288 in the hP2X7R flips further into the ATP-binding pocket to facilitate binding, Phe288 in the rP2X7R is positioned in a way that is predicted to disfavour interactions with these antagonists.

C23, C40 and C60 all contain an N-methyl amide group in their structure, with a prominent NO_2_ group at one end and a bulky ring structure at the opposite end (Table 1), thus suggesting that they represent a class of new hP2X7R antagonists with a structurally conserved pharmacophore. Preliminary studies show that replacement of the NO_2_ group in C40 with NH_2_ retains activity, whereas substitution with CH_3_ reduces inhibition of hP2X7R-mediated Ca^2+^ responses (data not shown). This suggests that nitrogen is a vital component of the compound structure which is possibly required for hydrogen bonding with residues such as Gln143 in the ATP-binding pocket (Fig. 7). A compound containing a benzene ring structure in replacement of the bulky ring structure in C23 and C40 is ineffective in inhibiting hP2X7R-mediated Ca^2+^ responses (data not shown), implying that the bulky ring structure is pivotal in interacting with the hP2X7R, particularly Tyr288. Furthermore, assessment of the compound structures predicts the central common moiety is a relatively flexible linker, allowing antagonists to adopt a bent configuration to facilitate interactions with the receptor (Fig. 7). Finally, all three compounds identified in this study preferentially inhibit the hP2X7R over the rP2X7R (Fig. 1D–G). Such striking species specificity has been repeatedly reported for known hP2X7R antagonists such as KN62 [19], AZ11645373 [40] and SB203580 [31]. Examination of ligand-receptor interactions has led us to hypothesise that the species specificity of C23, C40 and C60 arises from structural differences in the ATP-binding pocket such as Tyr288. This residue in the hP2X7R flips further into the ATP-binding pocket and its orientation is expected to facilitate, whereas Phe288 in the rP2X7R is positioned in a way that is predicted to disfavour interactions with the antagonists, specifically the bulky ring structure discussed above (Fig. 7). Further site-directed mutagenesis studies will inform the importance of these parts in the receptor including Gln143 and Tyr288 in determining antagonist binding.

In summary, the present study identifies for the first time structurally novel hP2X7R antagonists using a structure-based approach combining virtual screening and functional testing. It reports the first P2X7R antagonist with preferential inhibition of the large pore formation without effect on the ion channel function.

## Author contributions

E.A.C., S.P.M. and L.H.J. conceived and designed the research. E.A.C. performed the experiments, E.A.C. and L.H.J. analysed the data. S.P.M. and C.W.F. contributed to intellectual inputs, E.A.C. S.P.M. and L.H.J. wrote and revised the manuscript.

## Conflicts of interest

The authors state no conflict of interest.

## Figures and Tables

**Table 1:** Summary of chemical and pharmacological properties of novel hP2X7R antagonists.

Compound ID	ZINC12 database ID	Chemical structure	IC_50_ (μM) (Ca^2+^ response)	IC_50_ (μM) (current)	IC_50_ (μM) (dye uptake)
C23	ZINC 67825876		5.1 ± 0.3	0.35 ± 0.3	1.8 ± 0.9
C40	ZINC 58368839		4.8 ± 0.8	1.2 ± 0.1	1.0 ± 0.1
C60	ZINC 09315614		3.2 ± 0.2	≫100	0.8 ± 0.2
